# Evidence of self-care tooling and phylogenetic modeling reveal parrot tool use is not rare

**DOI:** 10.1016/j.isci.2025.112156

**Published:** 2025-03-04

**Authors:** Amalia P.M. Bastos, Scott Claessens, Ximena J. Nelson, David Welch, Quentin D. Atkinson, Alex H. Taylor

**Affiliations:** 1 Department of Psychological & Brain Sciences, Johns Hopkins University, Baltimore, MD, USA; 2 School of Psychology, University of Auckland, Auckland, New Zealand; 3 School of Biological Sciences, University of Canterbury, Christchurch, New Zealand; 4 School of Computer Science, University of Auckland, Auckland, New Zealand; 5 ICREA, Pg. Lluís Companys 23, Barcelona, Spain; 6 Institute of Neuroscience, Universitat Autònoma de Barcelona, Barcelona, Spain

**Keywords:** Exotic species behavior, Animals, Animal science

## Abstract

Putatively rare behaviors like tool use are difficult to study because absence of evidence can arise from a species’ inability to produce the behavior or from insufficient research. We combine data from digital platforms and phylogenetic modeling to estimate rates of tool use in parrots. Videos on YouTube revealed novel instances of self-care tooling in 17 parrot species, more than doubling the number of tool-using parrots from 11 (3%) to 28 (7%). Phylogenetic modeling suggests 11–17% of extant parrot species may be capable of tool use and identifies likely candidates. These discoveries impact our understanding of the evolution of tool use in parrots, revealing associations with relative brain size and feeding generalism and indicating likely ancestral tool use in several genera. Our findings challenge the assumption that current sampling efforts fully capture the distribution of putatively rare animal behaviors and offer a fruitful approach for investigating other rare behaviors.

## Highlights

• Tool use is thought to be a rare phenomenon in parrots
• Videos on a social media platform reveal self-care tooling in 17 new parrot species
• Phylogenetic modeling predicts further tool-using parrot species
• Encephalization and feeding generalism predict tool use among parrots

## Graphical abstract

**Figure:**
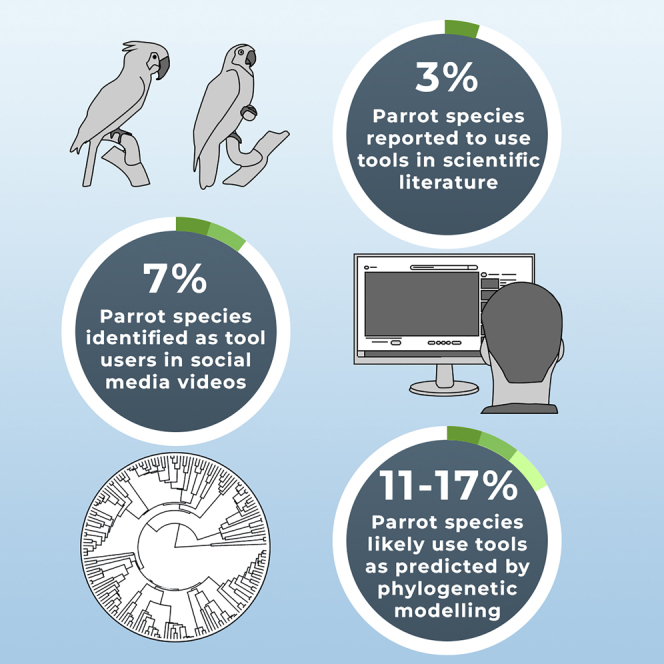

## Introduction

Our understanding of the evolution of animal behavior is built on the assumption that we have access to sufficient data.^1^^,^^2^^,^^3^ However, this is not always the case. Data on behaviors that are rare, fleeting, or otherwise difficult to observe are likely to be patchy and incomplete.^4^^,^^5^ Among species for which such behaviors have not been observed, it can be difficult to differentiate between cases in which the species is truly incapable of producing the behavior and cases in which the species is capable of producing the behavior but the behavior has not yet been observed. Such a distinction can be critical for drawing conclusions about the rarity and evolution of the behavior in question.

Comparative work on the evolution of tool use is a paradigmatic example of this issue. The initial discoveries of tool use in chimpanzees,^6^ birds,^7^ dolphins,^8^ and octopuses^9^ occurred decades after significant advances on other more easily measurable aspects of their biology. Since then, scholars have proposed a clear operational definition of tool use applicable to all species—the manipulation of an unattached object as an extension of the animal’s body to achieve a goal^10^—and have used the distribution of species meeting this definition to make various claims about the evolutionary drivers of tool use behaviors. For example, based on the observation that bird species with reported tool use tend to have larger brains, researchers have identified higher relative brain size as a likely precondition for tool using capabilities.^11^^,^^12^^,^^13^^,^^14^ These researchers argue that larger brains are better able to integrate visual and somatosensory information when innovating novel behaviors, such as tool use, in changing environments^15^^,^^16^ (but see the study by Kaplan^17^). Similarly, researchers have used existing reports of tool use in birds to debate the roles of generalist versus specialist feeding strategies in driving the evolution of tool use, with some arguing that feeding generalists require technical innovations to expand their dietary niche^15^^,^^18^^,^^19^ and others arguing that feeding specialists require technical innovations for extractive foraging of specific foods.^20^^,^^21^

However, before we can make claims of this kind, we need to know whether current research effort in the literature is sufficient for robust conclusions to be drawn about the evolution of tool use. In fact, evidence suggests that research effort is often systematically biased toward particular taxonomic groups, parts of the world that are easy to access, and species with life history traits that make them easier to study, such as larger distribution ranges and population sizes.^22^ This is a crucial limitation because insufficient observation may lead researchers to miss true instances of tool behaviors and, thus, draw premature conclusions about the evolutionary drivers and origins of tool use. Researchers are keenly aware of this problem and have attempted to deal with it in different ways, such as controlling for the number of scientific papers published on different species. However, previous work has not yet attempted to quantify and explicitly model the relationship between actual tool-using behavior and what is reported in the scientific literature. If more tool-using species exist than previously thought, this could have important implications for theories of the evolutionary drivers and origins of tool use and for our understanding of how rare this behavior actually is.

One potentially powerful method for quantifying actual rates of rare animal behaviors is by collating evidence of the behaviors from digital video platforms.^23^ With millions of videos posted every day, digital platforms like YouTube offer a valuable source of data on animal behavior. Digital video platforms have already been successfully used to uncover a variety of rare animal behaviors, including interspecies play in dogs,^23^ novel problem-solving behaviors in horses,^24^ and death-related behaviors in Asian elephants.^25^ By casting the net wider than the scientific literature, digital video platforms can provide an indication of the tool-using species that the literature might be missing.

**Figure 1:**
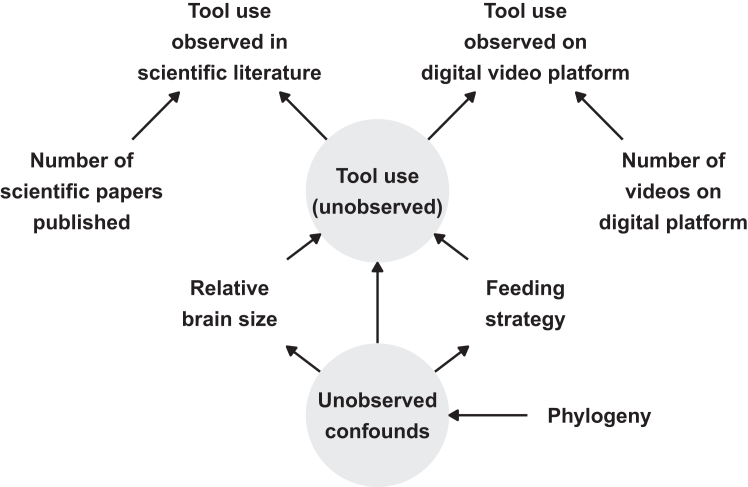
Causal model of observed tool use Directed acyclic graph of the causal relationships between observed tool use and other variables. Available scientific data on tool use is caused both by unobserved tool use presence and scientific research effort (i.e., number of publications). Available video data on tool use is caused both by unobserved tool use presence and video research effort (i.e., number of videos). According to theory, unobserved tool use presence should be caused by relative brain size (encephalization quotient) and feeding strategy (generalist vs. specialist). These variables all share unobserved confounds generated by shared phylogenetic history. Gray circles indicate unobserved variables.

Even after collating evidence from digital video platforms, some tool-users could *still* remain unobserved. One principled framework for identifying these unobserved species is to specify a causal model of the process that generates the observed data. We propose one such causal model in Figure 1. In this model, we assume that the presence of tool use in the scientific literature (or on digital video platforms) is caused by both unobserved tool use capabilities and the number of published studies (or the number of videos) for any given species. Tool users are more likely to be observed if they are well studied, but understudied tool users may go undetected. Furthermore, based on existing theories of the evolution of tool use,^12^^,^^13^^,^^14^^,^^15^^,^^16^^,^^18^^,^^19^^,^^20^^,^^21^ we propose that the unobserved tool use capabilities are additionally caused by relative brain size, feeding strategy, and shared phylogenetic ancestry. Expressing this causal model as a statistical model can suggest further species which are likely to be unobserved tool-users and, simultaneously, test existing theories of the evolutionary drivers of tool use without incorrectly assuming that absence of evidence is evidence of absence.

Here, we apply these approaches to tool use in the parrot order. We focus on tool use in parrots for a number of reasons. First, the scientific literature suggests that only a small proportion of extant parrot species (11 out of 398; 3%) use tools.^12^^,^^26^^,^^27^^,^^28^^,^^29^^,^^30^^,^^31^^,^^32^^,^^33^^,^^34^^,^^35^^,^^36^^,^^37^^,^^38^ Parrot tool use thus provides an ideal test case for examining how robust sampling is in the scientific literature. Second, parrots are popular as pets. Over 70% of all extant parrot species are bred in the aviculture industry and kept as pets worldwide,^39^^,^^40^^,^^41^^,^^42^^,^^43^^,^^44^^,^^45^ enabling us to leverage the power of digital video platforms to search for evidence of tool use.^23^ Third, detailed data on relative brain sizes,^46^^,^^47^^,^^48^^,^^49^^,^^50^^,^^51^ feeding strategies,^52^ and shared ancestry^53^ exist for parrots, allowing us to fit the statistical model implied by Figure 1 to the entire parrot order.

We first present the results from our video survey, in which we collated videos of tool use in parrots from a digital video platform. This survey reveals that a number of parrots not previously known to use tools are capable of self-care tooling. We then map these previously unidentified tool-using species onto the phylogeny of the parrot order and use a phylogenetic survival cure model to (1) rank further parrot species that are likely unobserved tool users and (2) re-examine key hypotheses regarding the evolutionary drivers and origins of tool use in parrots.

## Results

### Digital video platform reveals self-care tool use in additional parrot species

We surveyed the digital media platform YouTube for video evidence of tool use in parrots (see STAR Methods for detailed search criteria). In our search, we used the standard criteria for identifying tool use in the literature, defining “true” tool use behavior as the manipulation of an unattached object as an extension of the animal’s body to achieve a goal,^10^ while “borderline” tool use involved the use of an object that was still attached to a substrate.^54^

In total, we found 116 videos of 104 individuals from 25 parrot species performing behaviors that met the definition of either true tool use (100 videos of 89 individuals from 22 species) or borderline tool use (16 videos of 16 individuals from 7 species). All videos featured pet parrots in captive settings performing self-care tool use, specifically self-scratching. In 68 of these videos, owners did not appear to interact with the subjects. In 43 videos, there was potential human interaction, either from the owners being in close physical contact with the bird (e.g., bird perching on hand), talking to the bird, or handing it the tool (which occurred in only two videos). We could not establish the degree of human interaction in the remaining 5 videos, as sound had been removed or was substituted by music. None of the videos featured owners directly rewarding tool use behaviors with food. All borderline tool use cases were excluded from further analyses.

Of the 22 parrot species performing true tool use for self-care, 13 were represented in our video survey by two or more individuals over multiple independent observations. True tool use always involved the subject using an object for self-scratching (95 videos involved scratching the head and/or neck). The most common tool (53 videos) was a moulted feather. Human-made objects (e.g., pens, spoons, pieces of wood, cardboard) were also common. In 66 videos, parrots manipulated the tool while keeping their body still, rather than holding the tool still and moving toward it.

According to YouTube video descriptions and owner comments, 45 of the individuals performing true tool use for self-care were males and 18 were females. No sex information was provided for the remaining 26 individuals. As owners provided no information on whether sex had been established through genetic testing, and sexual dimorphism in parrots is rare,^55^^,^^56^ we could not typically ascertain if descriptions were accurate. It is unclear if the disproportionately large number of males in the sample is a consequence of owners more readily assuming their parrots are male when they have not been genetically tested, owners being more likely to own or film male parrots, or male parrots exhibiting more self-scratching behaviors than female parrots.

**Figure 2:**
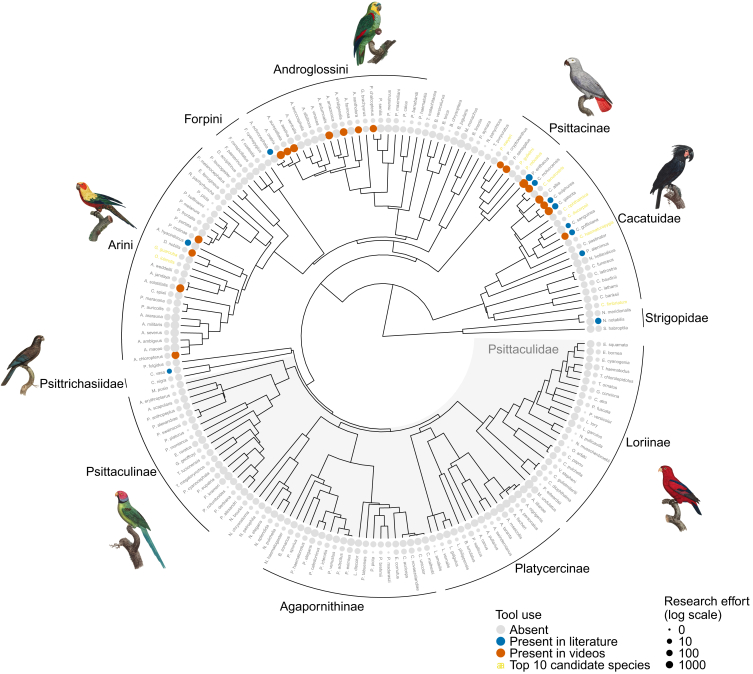
Results of video survey and phylogenetic survival cure modeling mapped onto a maximum clade credibility phylogeny of the parrot order Orange points in the inner ring indicate species observed in the video survey, with point size scaled by the number of videos for each species (note that three species observed only in the video survey are not present in the phylogeny due to a lack of genomic data: *Psittacara erythrogenys*, *Psittacus timneh*, and *Aratinga nenday*). Blue points in the outer ring indicate species observed in the scientific literature, with point size scaled by the number of papers published on each species. Yellow species names indicate the top ten most likely tool-using species from our phylogenetic survival cure model which were not observed in the scientific literature or the video survey. Total *n* = 174 species.

Figure 2 maps the findings from the video survey onto a maximum clade credibility phylogeny for the parrot order, plotted alongside species previously identified in the scientific literature. Before the video survey, 11 parrot species (3%) had been identified as tool users in the scientific literature. Across our video survey, we observed true tool use for self-care in 22 species, 5 of which overlapped with the scientific literature and 17 of which were novel species. All of the species identified in the video survey were cockatoos (*Cacatuidae*), Old World parrots (*Psittacinae*), or neotropical parrots (*Arinae*). The most common species in our survey, accounting for 41 videos from 37 individuals, was the green-cheeked conure (*Pyrrhura molinae*). In accordance with the scientific literature, the video survey found no evidence of tool use in any species of Psittaculidae, despite this family containing some of the most commonly kept pet species, including lovebirds, lorikeets, and Asian parakeet species. Combining both the video survey and the scientific literature, we can thus identify 28 total parrot species (7%) capable of true tool use as defined by Shumaker et al.,^10^ compared to the 11 previously reported.

The identification of self-care tooling behaviors in several new parrot species in our video survey increases the extent to which phylogeny can explain the distribution of a cognitive capacity for tool use in the parrot order. We estimated the phylogenetic signal (Pagel’s λ) of tool use using both the pre-video-survey and post-video-survey data. Pagel’s λ varies between 0 and 1, where 0 implies that the distribution of a trait across species is unexplained by phylogenetic relatedness and 1 implies that the distribution of a trait across species is fully explained by phylogeny. Using the evidence of tool use from the scientific literature alone (pre-video-survey data; 11 tool-using species), we estimated an average posterior Pagel’s λ of 0.60 (95% credible interval [0.00 0.90]; total *n* = 174 species). This estimate was moderate-to-strong, but highly uncertain. In comparison, combining the evidence from both the literature and the video survey (post-video-survey data; 28 tool-using species) resulted in a stronger and more certain estimate of phylogenetic signal. With these data, we estimated Pagel’s λ = 0.65 (95% CI [0.50 0.77]; total *n* = 174 species). Thus, the results of our video survey increase the extent to which the distribution of tool use capabilities across parrot species can be explained by shared phylogenetic ancestry. This suggests that we can potentially use phylogenetic information, along with other variables, to identify further parrot species that are capable of tool use but have remained undetected.

### Phylogenetic survival cure modeling identifies further candidate tool users

If we combine evidence from both the literature and our video survey, 28 parrot species in total have demonstrated a capacity for some form of tool use, whether that be self-care tool use in captivity, tool use in a wild foraging context, or other tool behaviors that fit the criteria for true tool use.^10^ By analyzing these observations alongside data on relative brain size, feeding strategy, and phylogenetic ancestry, we can (1) identify which parrot species without recorded evidence of tool use are most likely to have a capacity for tool use that has as yet gone unobserved, and (2) test theories about the evolutionary drivers and origins of technical intelligence in the parrot order. To this end, we fitted a Bayesian phylogenetic survival cure model to the data on parrot tool use from both the literature and our video survey.

Survival cure models,^57^ also known as split population models,^58^ are used to analyze the time to some event of interest with the added assumption that a certain proportion of the population will never experience the event, no matter how long they are measured for. These models have been used to analyze a variety of right-censored outcomes, from cancer relapse^57^ to criminal recidivism.^58^ The data are right-censored because some individuals will have experienced the event when they are measured (e.g., disease onset, return to prison) while others will have not experienced the event. For those who have not experienced the event, this may be because (1) the event has not happened to them yet or (2) the event will never happen to them. Survival cure models treat these two processes separately.

Our tool use problem has the same features. We are modeling a time-to-event; specifically, the amount of “time” (i.e., observation opportunities measured as the number of published papers or videos) until the capacity for tool use is identified in some form of tool use behavior. This is right-censored data because many species will not have had tool use identified when we measure them. Moreover, we can assume that a certain proportion of the population will never experience the event—that is, they do not have the capacity for tool use and so we will never observe tool use behavior no matter how long we measure them for.

In our model, we infer the tool-using status of each species by allowing each species to have their own probability of being a non-tool-user. Following our causal model (Figure 1), we predict these probabilities based on feeding strategy, encephalization quotient, and phylogenetic history (see STAR Methods for full model). The model additionally takes research effort into account by allowing that, among species for which tool use is unobserved, all else being equal those with fewer published papers and fewer video search hits have a higher probability of being undetected tool users (Figure S1).

We found that this phylogenetic survival cure model was able to adequately distinguish between species with and without evidence for tool use, with an area-under-the-curve classification statistic of 0.95 (Figure S2). To further estimate the accuracy of the model’s predictions, we also used a leave-one-species-out approach with known tool users. For each of the 25 tool-using species that were represented on the phylogeny and for which we had brain size and genomic data (we lacked data for three tool-using species), we fitted the model to a modified dataset which set tool use to be absent for the target species in both the scientific literature and the video survey. Across 25 cross-validation models, 18 models (72%) continued to predict the target species as having a median posterior probability of tool use that was within the range of all other tool users. This classification rate was greater than the baseline classification rate of 26% for species without evidence of tool use in the full model (38 of 149 species without evidence of tool use had a median posterior probability of tool use that was within the range of the tool-using species). Together, the area-under-the-curve statistic and the leave-one-species-out approach suggest that the model is able to adequately classify known tool users, with some error.

**Figure 3:**
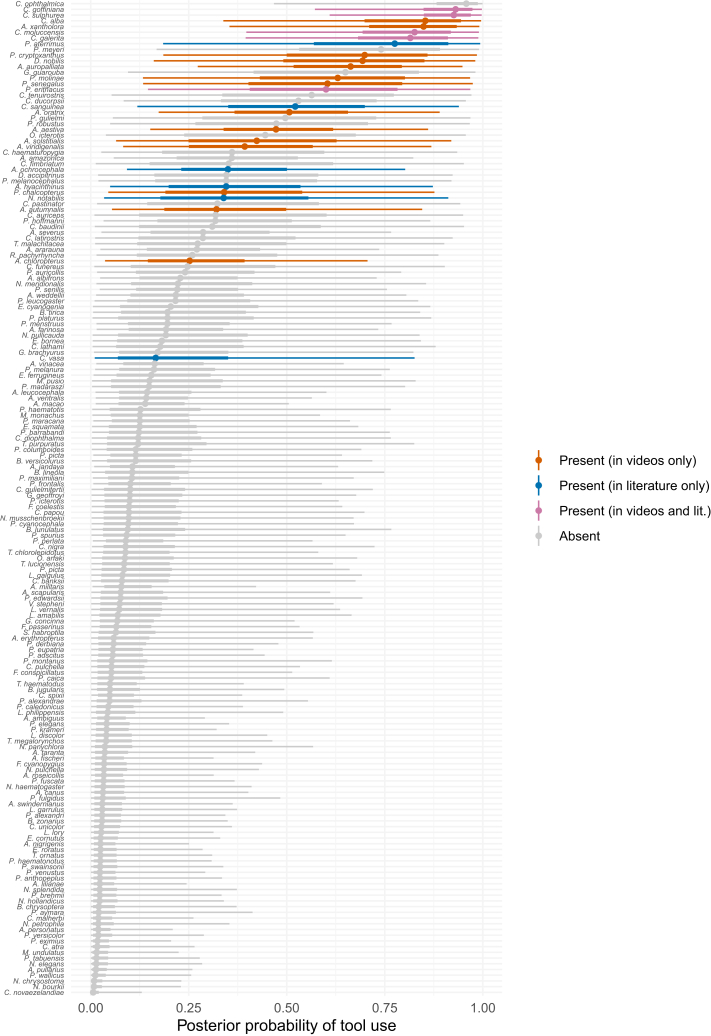
Posterior predicted probabilities of tool use for each species from our phylogenetic survival cure model Points are posterior medians and lines are 50% and 95% credible intervals. Total *n* = 174 species.

**Figure 4:**
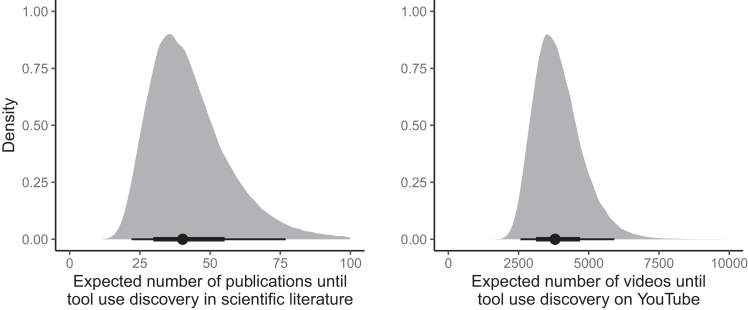
Expected number of published papers and videos until tool use discovery, according to the survival component of the phylogenetic survival cure model Densities are full posterior distributions, points are posterior medians, and lines are 50% and 95% credible intervals.

Figure 3 visualizes the ranked posterior probabilities of tool use from the phylogenetic survival cure model for all parrot species. As expected, the known tool users are ranked toward the top of this list. However, several “tool use absent” species also rank highly on the list, despite not being identified as tool users in the scientific literature or in our video survey. In fact, according to the model, the most likely tool user is a species for which tool use is unobserved in our data: the blue-eyed cockatoo (*Cacatua ophthalmica*). This species is endemic to Papua New Guinea and is relatively understudied, with only 6 published papers and 596 video search hits, which is fewer than the model expects are necessary to discover tool use when it is present (Figure 4). This species is also found in the *Cacatua* genus, a clade containing several known tool users. This prediction makes sense given the high phylogenetic signal for tool use reported by the model (Figures S3 and S4). Beyond the blue-eyed cockatoo, other highly ranked species without observed evidence of tool use are the Meyer’s parrot (*Poicephalus meyeri*), the golden parakeet (*Guaruba guarouba*), the long-billed corella (*Cacatua tenuirostris*), the Solomons cockatoo (*Cacatua ducorpsii*), the red-fronted parrot (*Poicephalus gulielmi*), the Cape parrot (*Poicephalus robustus*), the yellow-eared parrot (*Ognorhynchus icterotis*), the red-vented cockatoo (*Cacatua haematuropygia*), and the gang-gang cockatoo (*Callocephalon fimbriatum*). Figure 2 plots these species on the parrot phylogeny, using the top ten highest ranked species without observed evidence of tool use as an arbitrary cutoff for visualization purposes.

The posterior probabilities shown in Figure 3 are estimated with uncertainty, so it is difficult to “identify” any particular species as having an undetected capacity for tool use. Nevertheless, taking the sum of all the posterior probabilities for the 149 species without observed evidence of tool use, we can estimate that around 26 of those species are likely to be undetected tool users (median sum of probabilities = 25.68, 95% CI [15.15 41.33]). When combined with the species known to use tools, this implies that between 11% and 17% of extant parrot species may be tool users.

### Implications for the evolutionary drivers and origins of tool use

**Figure 5:**
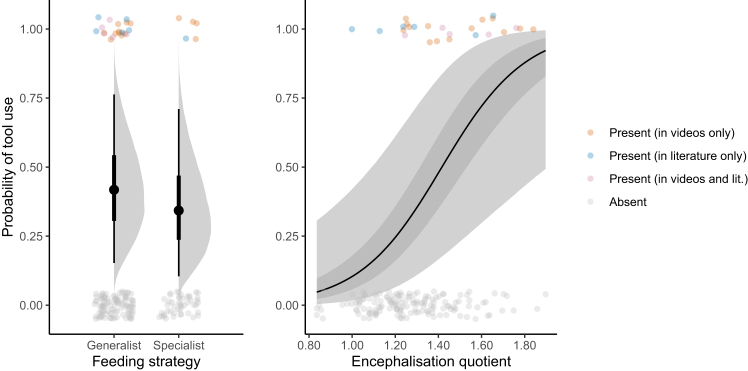
Posterior predictions for the effects of feeding strategy and encephalization quotient on the probability of tool use from the phylogenetic survival cure model In the left plot, points and lines represent posterior medians and 50% and 95% credible intervals, with densities representing full posterior distributions. In the right plot, the line and shaded areas represent the posterior median regression line with 50% and 95% credible intervals. In both plots, individual species are colored according to the presence/absence of tool use in the video survey and the scientific literature. Total *n* = 174 species, generalist *n* = 121 species, specialist *n* = 53 species.

The predicted probabilities from our phylogenetic survival cure model have implications for inferences about the evolutionary drivers and origins of tool use in the parrot order. Regarding the drivers of tool use hypothesized in Figure 1, the phylogenetic survival cure model revealed that encephalization quotient strongly positively predicted the probability of tool use (median posterior log odds slope = 1.12, 95% CI [0.39 2.00]; total *n* = 174 species; Figure 5). This helps explain the ranking in Figure 3: the blue-eyed cockatoo has the largest relative brain size in the dataset. We also found that feeding generalist species were slightly more likely to be tool users, though the posterior difference between generalists and specialists was quite uncertain (median posterior log odds difference = 0.33, 95% CI [−1.13 1.76]; total *n* = 174 species). These results from the survival cure model differed from the results of models fitted to pre-video-survey and post-video-survey data without the survival cure component, which found no effect of relative brain size and no difference between feeding strategies, respectively (Figure S5).

Regarding the origins of tool use, we fitted exploratory ancestral state reconstruction models to the pre-video-survey data, the post-video-survey data, and the predicted probabilities from the phylogenetic survival cure model. The discoveries from our video survey and from our phylogenetic modeling increased the likelihood that the capacity for tool use was present in the most recent common ancestors for several parrot genera. These include the most recent common ancestor of amazon parrots native to the Americas (*Amazona*), the most recent common ancestor of the true white cockatoos and corellas found in South East Asia and Australasia (*Cacatua*), the most recent common ancestor of the kea and the kākā from New Zealand (*Nestor*), and the most recent common ancestor for the *Poicephalus* genus native to Africa (Table 1; Figures S6–S8).

**Table 1:** Estimated probabilities of tool use for most recent common ancestors of several parrot genera

Genus	Pre-video-survey	Post-video-survey	Survival cure probabilities
*Amazona*	0.06, 95% CI [0.01 0.17]	0.21, 95% CI [0.04 0.66]	0.48, 95% CI [0.05 0.84]
*Cacatua*	0.08, 95% CI [0.03 0.20]	0.17, 95% CI [0.03 0.45]	0.81, 95% CI [0.23 0.99]
*Nestor*	0.11, 95% CI [0.05 0.29]	0.21, 95% CI [0.12 0.37]	0.53, 95% CI [0.29 0.66]
*Poicephalus*	0.06, 95% CI [0.02 0.11]	0.19, 95% CI [0.12 0.41]	0.72, 95% CI [0.16 0.90]

Probabilities estimated using exploratory ancestral state reconstruction models fitted to the pre-video-survey data, post-video-survey data, and predicted probabilities from the phylogenetic survival cure model. Total n = 174 species.

## Discussion

Since the earliest anecdotes of parrots using tools by Wallace in the 1880s^59^ and more systematic anecdotal reports in the 1970s,^60^ only 11 parrot species (3% of all extant parrots) have been documented as tool users in the scientific literature. Our study collated data from a digital video platform and revealed capabilities for self-care tool use in 17 additional parrot species, more than doubling the overall count of tool-using parrots to 28 species (7%). These species consisted of cockatoos (*Cacatuidae*), Old World parrots (*Psittacinae*), and neotropical parrots (*Arinae*). Beyond the video survey, the strong phylogenetic signal in our dataset allowed us to use phylogenetic information, along with other variables, to infer the unobserved probabilities of tool use across the parrot order. Our phylogenetic survival cure model incorporated information on phylogenetic history, research effort, relative brain size, and feeding specialization to rank parrot species that were most likely to be undetected tool users. The sum of probabilities from this model implied that between 15 and 41 of the species without evidence of tool use are likely to have an unobserved capacity for tool use, suggesting that the true proportion of tool users in the parrot order may be as high as 17%.

All instances of tool use in our video survey met the established criteria for tool use in the literature: the manipulation of an unattached object as an extension of the animal’s body to achieve a goal.^10^ However, a striking feature of the dataset is that the 17 additional species identified in the video survey were exclusively observed using tools for self-scratching. While parrots in the wild use tools to achieve a variety of goals, including extractive foraging and courtship,^34^^,^^35^^,^^38^^,^^61^ parrots in captivity rarely ever require tools in an environment where food is readily available and there is little or no competition for mates. Instead, the most commonly observed form of tool use in captive parrots is self-scratching. It could be argued that self-scratching is a more cognitively simple form of tool use than extractive foraging as it is egocentric, meaning that the tool is directed toward oneself rather than an external object. Nevertheless, self-scratching meets the definition for a more complex type of embodied tool use known as “tooling”, the deliberate generation of a mechanical interface by using an object to manipulate another target or surface.^62^ In our dataset, most self-scratching tooling instances involved manipulating the tool while keeping the body still, suggesting that these were goal-directed, deliberate movements by individuals. Moreover, the fact that we found strong phylogenetic clustering of self-scratching tool use from the video survey with other examples of tool use from the literature supports a common underlying cognitive mechanism. In line with this, some of the species of parrots that use tools for self-scratching in captivity also use tools for other purposes in the wild.^30^^,^^31^^,^^34^^,^^38^ For these reasons, we believe that self-care tool use in captivity is no less indicative of a cognitive capacity for tool use than other tool behaviors in the wild or in captivity. Indeed, if this is not the case, then the field will need to re-evaluate current working definitions of true tool use and their implications for animal cognition. This justifies our use of self-scratching, alongside other wild observations of tool use, to make claims about the evolutionary drivers and origins of technical intelligence in the parrot order.

Our data raise the question of why such a wide range of parrot species exhibit self-scratching tool use behaviors in captive environments. It is possible that these self-care behaviors emerge in captivity because pet parrots are kept with few or no conspecifics, and therefore must innovate tool use to preen inaccessible body areas, such as their heads and necks, which would have been otherwise preened by flock members. This may indicate that human interaction and allopreening in the absence of a conspecific is insufficient for pet parrots, as suggested by studies on welfare and chronic stress in lone captive parrots.^63^^,^^64^^,^^65^^,^^66^ Beyond social isolation in captivity, other factors that reduce preening efficacy by an individual might also lead to the innovation of self-care tooling: for example, a captive kea housed with conspecifics in a naturalistic environment was shown to have innovated the use of a pebble tool for self-care likely as a consequence of a missing upper mandible.^31^ These behaviors might therefore be innovated by individuals when ecologically necessary. Critically, however, a capacity to innovate a behavior such as tool use must exist in a species in order to manifest in any environment, whether that is captive or in the wild. The fact that only a subset of parrot species are innovating self-care tooling in response to their captive setting and that self-care tooling is predicted by phylogenetic history and encephalization strongly suggests that self-scratching indicates a capacity to produce tool use, which may be expressed in different ways in captive and wild settings. Interestingly, at present there is no evidence to suggest that parrots exhibit self-scratching tool use in the wild. This could reflect a true absence of this behavior in wild parrots, which may not experience the same environmental pressures that lead to the emergence of self-scratching in captivity. On the other hand, it could also reflect a false negative, with self-scratching occurring sufficiently infrequently or inconspicuously in wild populations that it remains unobserved or unreported.

Our findings have a number of important implications. First, our findings suggest that current research effort in the scientific literature is insufficient to capture the real world occurrence of parrot tool use. If the scientific literature had sampled the natural world sufficiently, we would expect to see close correspondence between those species reported as tool users in the literature and those species the public have filmed performing tool use. Instead, we discovered a large discrepancy between these two data sources, both in the prevalence of tool use and the species identified, likely due to the numerous difficulties inherent to conducting observations in the wild. This raises the possibility that other rare behaviors remain yet to be discovered in the scientific literature.

Second, in terms of the evolution of tool use in parrots, our study challenges a key assumption made in the literature to date: that only a minority of parrots are tool users.^30^^,^^31^^,^^34^^,^^37^^,^^38^ The paucity of evidence for tool use across parrots in the literature initially implied that the capacity for tool use may have evolved independently in different parrot species. Our discovery of the widespread distribution of tool use across the parrot phylogeny, along with the strong phylogenetic signal in this expanded dataset, challenges this and suggests that, at least for some parrot clades, the capacity for tool use might be a homologous trait that has been evolutionarily conserved. Our exploratory ancestral state reconstruction analysis provides preliminary support for this hypothesis, revealing an increased probability of the capacity for tool use among the most recent common ancestors for the *Amazona*, *Cacatua*, *Nestor*, and *Poicephalus* genera. The complexity of tool use behavior that such a basic capacity might afford, and the conditions under which it emerges in the wild, are yet to be established. However, even at this preliminary stage, our analysis raises an alternative hypothesis for the observed tool use in *Cacatua*^30^^,^^38^ and *Nestor*,^26^^,^^27^^,^^28^^,^^29^^,^^31^^,^^34^ namely that tool behaviors have arisen due to the common ancestor having the capacity to use tools, rather than from independent evolution or behavioral innovation within species.

Third, our results support existing theories of the drivers of tool use. We found that encephalization was strongly positively related to the probability of tool use in our phylogenetic model, supporting previous theories linking relative brain size to increased tool innovation in birds^12^^,^^13^^,^^14^ and primates.^67^ We acknowledge that encephalization quotient is not a perfect measure due to measurement error and challenges with interpretation.^3^^,^^4^ However, encephalization quotient has much greater coverage across the parrot phylogeny than more fine-grained measures like whole neuron count,^68^ and there is no reason to think that measurement error would produce the consistent patterns across our study and prior work. To understand the causal mechanisms responsible for these relationships, we encourage further work on the specific neural correlates of technical intelligence in parrots, e.g., Cabrera-Álvarez and Clayton.^69^ In our phylogenetic model, we also found that tool use was somewhat more likely among feeding generalists compared to feeding specialists, although this difference was uncertain. This trend supports previous suggestions that increased cognitive abilities and technical innovation rates are required to expand a generalist species’ dietary niche.^18^^,^^20^^,^^70^ However, the trend contradicts theories linking tool use to dietary specialization, whereby species eating specific foods that require extractive foraging have higher cognitive ability and are especially prone to using tools.^20^

While missing data imputation is becoming more common in phylogenetic analyses,^71^ the important distinction between absence of evidence and evidence of absence has not been given as much attention. Our phylogenetic analysis provides one approach to this problem by distinguishing between true absences of tool use and absences of tool use due to a lack of research effort in the scientific literature or in videos. To achieve this, we explicitly modeled the measurement of the outcome variable along a research effort time series, such that species with lower research effort in the literature or in videos were likely to be censored. In line with our causal model, we also included relative brain size, feeding strategy, and phylogenetic history as predictors of unobserved tool use. We encourage researchers to test this model by directing future study efforts toward the parrot species with the highest probabilities of being undetected tool-users. Future work should also refine the causal model in Figure 1 to provide more certain estimates of tool use probabilities, either by including additional predictor variables or modeling further causes of measurement error in the taxonomic record, such as species abundance and geographic accessibility.^22^

In conclusion, we have shown that the scientific literature has insufficiently captured the full distribution of tool use in the parrot order. Our digital video survey identified novel observations of self-care tool use in several parrot species, more than doubling the number of known tool using parrot species from 11 to 28. Our phylogenetic model suggested that the true proportion of parrot tool users could be as high as 17% of all species in this order. These discoveries have implications for theories of the evolutionary drivers and origins of tool use in parrots. Beyond parrot tool use, the methods used in this study have the potential to be applied to other rarely observed behaviors, including tool use in other taxa,^72^ rhythmic entrainment in birds,^73^^,^^74^^,^^75^^,^^76^ teasing behaviors in primates,^77^ and tactical deception across all animals.^78^^,^^79^^,^^80^ We hope that these methods will continue to uncover a diverse array of ephemeral behaviors that have as yet gone undetected in the scientific literature.

### Limitations of the study

A potential concern with data originating from digital video platforms is its reliability.^23^^,^^81^^,^^82^^,^^83^^,^^84^^,^^85^ In particular, the self-care tool use instances detected in our video survey might be attributable to training or unintentional cueing by the birds’ owners. However, there was little evidence to suggest that the observations of self-scratching tool use in the video survey were merely unintentional accidents or explicitly trained behaviors. Individual parrots often used tools slowly and repetitively over long periods of time, even across multiple different videos, suggesting that their behavior was not random or accidental.^84^ Parrots preferentially employed self-scratching tools on areas of their body that were otherwise inaccessible, with 96% of all instances involving scratching of the head or neck, suggesting intentional tool use. For 60% of the species in the video survey, we found two or more videos of repetitive and sustained scratching by different individuals of the same species in separate households, suggesting that the manipulations were intentional and recurring events that did not represent unusual stereotypies of any single individual. Regarding the possibility of training or cueing from owners, over half of the videos contained no evidence of human interaction aside from filming the behavior. Humans only handed parrots their tools in two of the videos, and none of the videos featured owners directly rewarding tool use behaviors with food. It is unlikely that a range of owners would independently train parrots to scratch themselves with tools. Finally, the high levels of phylogenetic signal in our data provide strong evidence that the observations from our video survey reflect biologically endowed capacities for tool use rather than accidental or trained behaviors, which would likely appear uniformly across the phylogeny. Nevertheless, future work using data such as this should choose target behaviors carefully and examine videos for evidence of training or cueing.

## Resource availability

### Lead contact

Further information about resources should be directed to Scott Claessens (scott.claessens@gmail.com).

### Materials availability

This study did not generate new unique materials.

### Data and code availability

• All data have been deposited on GitHub and are publicly available as of the date of publication at https://doi.org/10.5281/zenodo.12732282.
• All original code has been deposited on GitHub and is publicly available as of the date of publication at https://doi.org/10.5281/zenodo.12732282.
• Any additional information required to reanalyze the data reported in this paper is available from the lead contact upon request.

## Acknowledgments

This project was made possible through the support of grants from the Templeton World Charity Foundation (A.H.T., X.J.N., TWCF-2018-0310, TWCF-2024-33225). The authors would like to thank Daniel Sol for providing feedback on a previous version of the manuscript.

## Author contributions

All authors contributed to the conceptualization of the paper; A.P.M.B., X.J.N., and A.H.T. developed the video search methodology; S.C., D.W., and Q.D.A. developed the statistical models and analyzed the data; All the authors wrote the manuscript and approved the final version for submission.

## Declaration of interests

The authors declare no competing interests.

## STAR★Methods

### Key resources table

**Table:** 

REAGENT or RESOURCE	SOURCE	IDENTIFIER
**Deposited data**		

Data from YouTube video survey	GitHub	https://doi.org/10.5281/zenodo.12732282
Additional data for parrot species	GitHub	https://doi.org/10.5281/zenodo.12732282

**Software and algorithms**		

R and Stan code	GitHub	https://doi.org/10.5281/zenodo.12732282

### Method details

#### Video searches and coding

Our video search was conducted on YouTube in July 2020. Searches were conducted manually by the first author over a month-long period, using the same IP address and not logged in with a YouTube user account. Search terms included “parrot using tool” and variants (e.g., “macaw using tool”, “lorikeet using tool”, “parakeet using tool”), “tool use in parrot”, “parrot tool use”, “parrot scratching itself” (included after we found several videos demonstrating self-care tool use in previous searches) and equivalent terms (e.g., “parrot preening itself”, “parrot grooming itself”, “parrot scratching”). For all species that did not display results including object manipulation or scratching behaviours, we also searched the species’ common name(s) + “tool use”, as well as the species’ common name(s) + “scratching”. We also searched for translations of the terms “parrot tool use” and “parrot scratching” in languages for all countries where bird ownership was reported as >5%,^86^ namely, Turkish, Czech, Polish, French, Italian, Dutch, German, Russian, Spanish, Portuguese, and Mandarin. Browser search histories were not cleared between searches.

When we found a relevant video, we also searched for similar content uploaded by the same person/channel. For each YouTube search conducted, we watched all relevant videos until we reached five consecutive videos that did not feature any parrots. At this point, we ended that search and initiated the next search. In line with previous recommendations,^23^ we planned to exclude any videos that consisted of four or more shots edited together so as to ensure the behaviours being observed were not edited or manipulated, but none of the videos obtained qualified for exclusion.

All videos featuring parrots manipulating objects were investigated for potential tool use or borderline tool use. We defined tool use as the manipulation of an unattached object as an extension of the beak or foot to achieve a goal towards another object, individual, or oneself.^10^ Borderline tool use was similarly defined, except that it involved the use of an object that was still attached to a substrate.^54^ For example, if individuals used a fallen feather or stick for self-scratching this was defined as tool use, but using one’s currently attached tail feathers or cage furnishings for the same purpose was defined as borderline tool use. Self-scratching had to involve slow and repeated movements of touching an object to one’s body (or, in the case of borderline tool use, rubbing repetitively against an attached object^84^).

All relevant videos were coded for video length, species, tool use presence (yes/borderline), tool use type (e.g., scratching, feeding), the object being used (e.g., feather, stick), tool use target, human interaction (talking or handing object to parrot, holding parrot), and the number of shots within each video. Our complete dataset also includes the name for each video, link, subject name, sex (as declared by owner, as most parrot species are not sexually dimorphic), publishing date, and dates found and coded.

Since YouTube is constantly growing and evolving in both its uploaded content and its proprietary recommendation algorithms, the same search strategy today would not return exactly the same videos as our search in July 2020. It is possible that some videos have since been deleted or more videos of parrot tool use have since been uploaded. However, this does not affect our conclusions about the number of parrot species found to use tools in our initial search.

#### Data for parrot species

We collected data for 194 parrot species (Figures S9–S11). We gathered feeding strategy data as a dichotomous variable (“generalist” or “specialist”) from the EltonTraits ecological database.^52^ As per the database, specialists were defined as species whose diet comprised at least 70% of a single food source. To calculate relative brain size, we collated data from the literature for all known body mass (g) and brain mass (g) values across parrots.^46^^,^^47^^,^^48^^,^^49^^,^^50^^,^^51^ For all species for which we obtained body and brain mass data, we calculated the encephalisation quotient (EQ) using the following formula^87^: $BrainWeight/\left(0.12*BodyWeight\left(\frac{2}{3}\right)\right)$. We found body mass and brain mass data for a total of 194 parrot species. This included all tool-using species in our video dataset, with the exception of three species: *Diopsittaca nobilis*, *Psittacara erythrogenys*, and *Coracopsis vasa*. For the latter, we used values for the closely related *Coracopsis nigra*. The other two species were excluded from the final dataset.

For modelling purposes, we coded research effort in both the scientific literature and the YouTube videos. For the scientific literature, we operationalised research effort as the number of papers published for each species’ Latin name up to and including the first paper containing tool use for that species. If no tool use had been identified in the scientific literature for a species, then we coded the total number of papers published to date. We used the scientific database Scopus for coding the number of published papers. For the videos, we coded research effort as the number of search hits for each species on YouTube. If tool use had been identified on YouTube, we estimated the number of search hits when the first video of tool use was published on YouTube, assuming linear growth of search hits since the inception of YouTube. If tool use had not been identified, we used the current number of search hits.

For phylogenetic data, we used the phylogenetic tool at www.birdtree.org ^53^ to compile 1000 posterior draws of phylogenetic trees for 174 of the 194 parrot species for which both EQ and genomic data exist. A single maximum clade credibility tree was generated from these posterior draws for visualisation purposes. In our analyses, we iterated over posterior draws of the phylogeny to account for phylogenetic uncertainty.

### Quantification and statistical analysis

#### Phylogenetic signal

We used the *fitDiscrete* function in the *ape* R package^88^ to calculate phylogenetic signal, for both the pre-survey and post-survey tool use data. We iterated the model over 100 posterior parrot phylogenies to incorporate phylogenetic uncertainty.

#### Causal model of tool use

To infer unobserved probabilities of tool use across parrots, we proposed a causal model of observed tool use (Figure 1). We assumed that observed tool use in the scientific literature and in the videos is caused by both the unobserved presence or absence of tool use and research effort, proxied by the number of papers published on a species and the number of videos published on a species. Tool users are more likely to be observed if they are well studied, but understudied tool users may go undetected. In addition, based on theory, we also assumed that unobserved tool use is caused by feeding strategy and relative brain size.^12^^,^^13^^,^^14^^,^^15^^,^^18^^,^^19^^,^^20^ Finally, we assumed that shared phylogenetic history causes unobserved confounding and non-independence in unobserved tool use, feeding strategy, and relative brain size across the parrot phylogeny.

#### Bayesian phylogenetic survival cure model

Given our proposed causal model, we constructed a statistical model to impute unobserved probabilities of tool use and test existing theories of the evolution of tool use in parrots. To understand the model, suppose that we have the following observed variables for parrot species i. For the scientific literature, we declare $N_{\text{Lit},i}$ as the number of papers published before and up to tool use identification for species i (or, if tool use has not been identified, the total number of papers published for species i) and $T_{\text{Lit},i}$ as a binary variable stating whether (1) or not (0) tool use has yet been observed in the scientific literature for species i. For the videos, we declare $N_{\text{Vid},i}$ as the number of videos published before and up to tool use identification for species i (or, if tool use has not been identified, the total number of videos published for species i) and $T_{\text{Vid},i}$ as a binary variable stating whether (1) or not (0) tool use has yet been observed in the videos for species i. Additionally, $F_{i}$ and ${\text{EQ}}_{i}$ are feeding strategy and encephalisation quotient values for species i and we have a phylogenetic distance matrix D that describes the patristic distances between all parrot species.

We assume that species i is a non-tool-user with some probability $p_{i}$. We also assume that tool use is identified in the scientific literature and the videos at constant rates $\lambda_{\text{Lit}}$ and $\lambda_{\text{Vid}}$ following exponential survival functions. Given these assumptions, we can then describe the different ways in which variables $N_{\text{Lit}}$ and $N_{\text{Vid}}$ can be distributed. Focusing on the scientific literature, if tool use has been observed $\left(T_{\text{Lit},i}=1\right)$, then the likelihood for $N_{\text{Lit},i}$ is:

(Equation 1) $$\mathrm{Pr}\left(N_{\text{Lit},i}|T_{\text{Lit},i}=1,p_{i},\lambda_{\text{Lit}}\right)=\left(1-p_{i}\right)\cdot \text{Exponential}\left(N_{\text{Lit},i}|\lambda_{\text{Lit}}\right)$$

On the other hand, if tool use has not yet been observed $\left(T_{\text{Lit},i}=0\right)$, there are two ways that the outcome variable could have been realised. First, the species could be a non-tool-user with probability $p_{i}$. Second, the species could be a tool-user with probability $\left(1-p_{i}\right)$ that has been censored and has not had its tool use measured yet. Together, then, the likelihood for $N_{\text{Lit},i}$ is:

(Equation 2) $$\mathrm{Pr}\left(N_{\text{Lit},i}|T_{\text{Lit},i}=0,p_{i},\lambda_{\text{Lit}}\right)=p_{i}+\left(\left(1-p_{i}\right)\cdot \text{ExponentialCCDF}\left(N_{\text{Lit},i}|\lambda_{\text{Lit}}\right)\right)$$

The Exponential CCDF function allows for the censored nature of the data. The same data generating process is assumed to underlie the videos.

We define the mixture likelihood SurvivalCure as the distribution above, with parameters p (the probability of being a non-tool-user) and λ (the rate of the exponential distribution). We use an Ornstein-Uhlenbeck Gaussian process^89^ to model phylogenetic covariance. Below, we specify the full model with priors:

(Equation 3) $$\begin{array}{c} T_{\text{Lit},i},N_{\text{Lit},i}∼\text{SurvivalCure}\left(\lambda_{\text{Lit},i},p_{i}\right)\\ T_{\text{Vid},i},N_{\text{Vid},i}∼\text{SurvivalCure}\left(\lambda_{\text{Vid},i},p_{i}\right)\\ \lambda_{\text{Lit},i}=1/\exp\left(\gamma_{\text{Lit}}\right)\;\\ \;\lambda_{\text{Vid},i}=1/\exp\left(\gamma_{\text{Vid}}\right)\;\;\\ \;\text{logit}\left(p_{i}\right)=\alpha_{\text{FEEDING}\left[i\right]}+\beta {\text{EQ}}_{i}+k_{\text{SPECIES}\left[i\right]}\\ \;\left(\begin{array}{l} k_{1}\\ k_{2}\\ \ldots \\ k_{n} \end{array}\right)∼\text{MVNormal}\left(\begin{array}{l} \left(\begin{array}{l} 0\\ 0\\ \ldots \\ 0 \end{array}\right),K \end{array}\right)\\ K_{ij}=\eta^{2}\;\exp\left(-\rho^{2}D_{ij}\right)\;\\ \gamma_{\text{Lit}},\gamma_{\text{Vid}},\alpha_{1,2},\beta ∼\text{Normal}\left(0,1\right)\;\\ \eta^{2},\rho^{2}∼\;\text{Exponential}\left(0.5\right)\; \end{array}$$

The priors for this model produce reasonable prior predictions of the probabilities of tool use for each parrot species (Figure S12), but a sensitivity analysis revealed that the ranking and posterior probabilities reported in the main text were robust to modifying these priors (Figure S13). We estimated the posterior distribution of this model using Hamiltonian Monte Carlo as implemented in Stan version 2.26.1.^90^ We ran the model for 4000 samples, with 2000 warmup samples, and iterated the model over 100 posterior parrot phylogenies to incorporate phylogenetic uncertainty. R-hat values and effective sample sizes suggested that the model converged normally. Trace plots are reported in Figure S14. We report equal-tailed credible intervals to describe the posterior distribution of this model in the main text.

To validate our method, we fitted the model to 100 simulated datasets with known parameters. The model was able to successfully recover those parameters (Figure S15). We also ran a leave-one-species-out exercise to ensure that we could accurately predict known tool users. We repeated this approach for each known tool user by setting observed tool use to zero. Cross-validation results are reported in the main text.

#### Ancestral state reconstruction

To determine whether the identification of novel tool-using species has implications for our understanding of the evolutionary origins of tool use in parrots, we fitted three exploratory ancestral state reconstruction models. We used the *ancThresh* function from the *phytools* R package,^91^ iterating the function over 100 posterior parrot phylogenies. This function estimates discrete ancestral states by assuming the evolution of a latent continuous variable following an Ornstein-Uhlenbeck process. We fitted this model to three different outcome variables: (*i*) presence vs. absence of tool use in scientific literature only, (*ii*) presence vs. absence of tool use in literature and/or videos, and (*iii*) the median predicted probabilities of tool use from the phylogenetic survival cure model.

#### Reproducibility

All analyses were conducted in R v4.2.1.^92^ Visualisations were produced using the *ggtree*,^93^ *ggplot2*,^94^ and *cowplot*^95^ packages. The manuscript was reproducibly generated using the *targets*^96^ and *papaja*^97^ packages. Code to reproduce all analyses and figures can be found here: https://github.com/ScottClaessens/phyloParrot.
